# Associations of six adiposity indices with incident back pain and the mediating role of the triglyceride-glucose index: prospective findings from the UK biobank

**DOI:** 10.3389/fnut.2026.1837601

**Published:** 2026-08-05

**Authors:** Jingqian Li, Xuefei Han, Yunqian Li, Yiyin Gao, Jiacheng Ding

**Affiliations:** 1 The Second Hospital of Jilin University, Changchun, China; 2 Jilin University, Changchun, China; 3 Department of Neurosurgery, The First Hospital of Jilin University, Changchun, China

**Keywords:** adiposity, back pain, prospective observational study, triglyceride-glucose, UK biobank

## Abstract

**Background:**

Back pain is now recognized as a major contributor to global disability. While the correlation between BMI and back pain risk has been confirmed, there is a lack of research on the association between obesity indices that accurately reflect fat distribution and back pain risk. Additionally, no studies have investigated whether the association is modified by any genetic factor, and remains unclear about the meditating role of triglyceride glucose (TyG).

**Methods:**

This prospective study involved participants from the UK Biobank without back pain at baseline. Cox regressions were utilized to analyze the relationships between BMI, waist circumference, hip circumference, waist-to-hip ratio (WHR), waist-to-height ratio (WHtR), and A Body Shape Index (ABSI) with back pain risk. The interactions between these obesity indices and genetic risk were also examined. Additionally, we investigated the meditating role of TyG for further understanding.

**Results:**

The multivariate Cox regression indicated that high levels of obesity indices were significantly correlated with back pain [BMI: hazard ratio (HR) = 1.45, 95% confidence interval (CI): 1.40–1.49, *p* < 0.001; Waist circumference: HR = 1.38, 95% CI: 1.34–1.42, *p* < 0.001; Hip circumference: HR = 1.36, 95% CI: 1.32–1.41, *p* < 0.001; WHR: HR = 1.30, 95% CI: 1.25–1.35, *p* < 0.001; WHtR: HR = 1.50, 95% CI: 1.45–1.55, *p* < 0.001; ABSI: HR = 1.14, 95% CI: 1.10–1.18, *p* < 0.001]. No significant multiplicative or additive interactions were found between any obesity indices and genetic susceptibility. Moreover, we found that TyG mediated the relationship between obesity indices and back pain incidence, with mediation proportions of 8.9, 10.2, 10.6, 19.8, 9.1, and 31.3% for high BMI, high waist circumference, high hip circumference, high WHR, high WHtR and high ABSI, respectively.

**Conclusion:**

Obesity is significantly and positively related to back pain risk with TyG playing a partial mediating role, but no significant interaction was observed between obesity and genetic susceptibility. As a result, focusing on obesity and metabolic dysfunction in obese individuals is crucial for reducing the risk of back pain.

## Introduction

Back pain is now recognized as a major contributor to global disability, with 37% median annual prevalence in adult population. The incidence reaches peak in middle age and is greater in female individuals (1). However, people from all age groups suffer from back pain, with an estimated lifetime incidence of 84% (2). In addition, the direct medical costs are considerable, which is even higher because of indirect costs associated with lost productivity. However, the exact cause for back pain is now unclear (3), so identifying modifiable risk factors associated with back pain may help inform prevention strategies.

It has been well documented that obesity contributes to multiple chronic conditions, such as cardiovascular disease (4), diabetes (5, 6), musculoskeletal diseases (7, 8). Some previous studies suggested a significant linkage between obesity and back pain risk. British Age Longitudinal Study found that lower BMI was related to decreased back pain risk (9). Another longitudinal analysis of Osteoarthritis Initiative participants suggested the connection of elevated BMI greater back pain risk (10). However, although BMI has been widely accepted for evaluating obesity and body composition, it solely includes overall body weight relative to height. As a consequence, BMI fails to distinguish different components including fat and muscle, nor does it provide information on the allocation and of accumulation fat (11). Other indicators including Waist circumference, hip circumference, WHR, ABSI and WHtR could reflect the distribution of fat more accurately than BMI. But there are fewer studies shedding light on their predicting value in back pain.

Emerging evidence establishes insulin resistance (IR) as a central pathophysiological mechanism bridging obesity-related metabolic dysregulation (impaired glucose tolerance, dyslipidemia, and chronic inflammation) to multisystem comorbidities (12, 13). While the hyperinsulinemic-euglycemic clamp remains the gold standard for quantifying IR, its clinical utility is constrained by labor-intensive protocols and substantial resource requirements, rendering it impractical for population-scale epidemiological studies (14–16). In contrast, the triglyceride-glucose (TyG) index—calculated through a simple logarithmic formula [ $\mathrm{TyG}\;\text{index}=\ln\frac{\mathrm{TG}(\mathrm{mg}/\mathrm{dL})\times \mathrm{FBG}(\mathrm{mg}/\mathrm{dL})}{2}$ ]—has emerged as a validated surrogate marker of IR, demonstrating superior feasibility for large-scale clinical applications (17, 18). Recent epidemiological investigations have illuminated the TyG index’s association with skeletal health outcomes. Analysis of the U. S. NHANES cohort (*n* = 6,752 adults aged 50–80 years) demonstrated a negative dose–response relationship between TyG index elevation and lumbar spine bone mineral density (*β* = −0.032 g/cm^2^ per TyG unit, *p* < 0.001) (19). Similarly, another study involving postmenopausal women demonstrated that elevated TyG levels were related to a reduced risk of osteoporosis (20). Despite these advances, a critical knowledge gap persists regarding the TyG index’s potential linkage to axial skeletal morbidity—particularly chronic back pain, a prevalent yet etiologically complex condition in metabolically compromised populations.

In addition, genetic factors have been shown to be associated with an elevated risk of back pain (21–23), which may exacerbate the negative effects of obesity on musculoskeletal health, leading to more severe and persistent back pain. Polygenic risk scores (PRS) were calculated based on the number of genetic risk variants carried by individuals and their association strength with back pain. This method provided a quantitative assessment of genetic susceptibility, allowing the estimation of lifetime genetic risk on disease development (24). Significantly, it remains uncertain whether obesity can participate in interactions with genetic predisposition to ultimately influence back pain risk. Here we intended to assess the linkage between obesity and back pain utilizing dataset from the UK Biobank. We also shed light on the mediating effect of TyG, the potential joint effects of obesity and genetic susceptibility with back pain risk.

## Methods

### Study population

This study was based on the UK Biobank (UKB), a large-scale prospective cohort that recruited more than 500,000 participants from 22 assessment centres across England, Scotland, and Wales between 2006 and 2010, with follow-up ongoing. Extensive phenotypic and genotypic information was collected from participants through touchscreen questionnaires, physical examinations, and biological sample analyses. Details of the study design and data collection procedures for the UKB have been described in previous publications (25, 26). The study was approved by both the National Health Service National Research Ethics Service (ref: 11/NW/0382) and the Institutional Review Board of Tulane University (study number: 2018–1872). All research conducted within the UKB was approved by the North West Multi-Centre Research Ethics Committee, and all participants provided written informed consent. This study was conducted in accordance with the Declaration of Helsinki.

This study initially included 501,940 participants. The following exclusion criteria were then applied sequentially: pregnant at baseline (*n* = 370), a diagnosis of back pain before baseline (*n* = 47,745), missing data on BMI (*n* = 2,719), waist circumference (*n* = 81), hip circumference (*n* = 29), triglycerides (TG) (*n* = 29,184), glucose (*n* = 35,809), and polygenic risk score (*n* = 4,332). In addition, we excluded participants having BMI less than 18.5 kg/m^2^ (*n* = 2,025), and finally 379,646 participants from UK Biobank were included. The selecting process is presented in Supplementary Figure 1.

### Outcome assessment

Diagnostic information was obtained by linking electronic health data to National Health Service (NHS) records. Data on non-cancer conditions were derived from the First Occurrence fields provided by the UK Biobank. The First Occurrence fields integrate primary care data (Read v2/v3), hospital inpatient data, death registry data, and self-reported condition codes recorded at the baseline or subsequent assessment centre visits, all mapped to 3-character ICD-10 codes. The ICD codes related to back pain are listed in Supplementary Table 1. Follow-up began at the baseline visit and continued until the first occurrence of the relevant outcome, death, loss to follow-up, or the end of the follow-up period, whichever came first. During follow-up, dates and causes of death were obtained from death certificates held by the NHS Information Centre for England and Wales and the NHS Central Register Scotland for Scotland. Dates and causes of hospitalization were ascertained through linkage to Hospital Episode Statistics for England and Wales and the Scottish Morbidity Record 01 (SMR01) for Scotland. Disease outcome follow-up was censored at September 2023, and mortality data were censored at July 2024.

### Calculation of indexes

The indicators of obesity included BMI, waist circumference, hip circumference, waist-to-hip ratio (WHR), waist to height ratio (WHtR) and A Body Shape Index (ABSI). The calculation formulas for BMI, WHR, WHtR, and ABSI are as follows: $\mathrm{BMI}=\frac{\text{Weight}\;(\mathrm{kg})}{\text{Height}\;{\left(m\right)}^{2}}$ , $\mathrm{WHR}=\frac{\text{Waist circumference}\;(\mathrm{cm})}{\mathrm{Hip}\;\text{circumference}\;(\mathrm{cm})}\;$ , $\text{WHtR}=\frac{\text{Waist circumference}\;(\mathrm{cm})}{\text{Height}\;(\mathrm{cm})}$ , and $\text{ABSI}=\frac{\text{Waist circumference}\;(\mathrm{cm})}{\mathrm{BMI}\;{\left(\mathrm{kg}/m^{2}\right)}^{2/3}\times \text{Height}\;{\left(m\right)}^{1/2}}\;$ (27). According to WHO recommendations, BMI was categorized as low (18.5 to 25.0 kg/m ^2^), medium (25.0 to 30.0 kg/m ^2^), and high (> = 30.0 kg/m ^2^) (28). Waist circumference was categorized as low (<94 cm for male; <80 cm for female), medium (94 to 101.9 cm for male, 80 to 87.9 cm for female), high (>102 cm for male; >88 cm for female) (29). WHtR circumference was categorized as low (<0.5), medium (0.5 to 0.6) and high (>0.6) (30). Hip circumference, WHR and ABSI were divided into low (Q1), medium (Q2, Q3), and high (Q4). Referring to previous literature (31, 32), the calculation formula of TyG is as follows: $\mathrm{TyG}\;\text{index}=\ln\frac{\mathrm{TG}(\mathrm{mg}/\mathrm{dL})\times \mathrm{FBG}(\mathrm{mg}/\mathrm{dL})}{2}$ .

### Genotyping and weighted polygenic risk scores

PRS were computed as the weighted sum of individual genetic variant dosages, with weights corresponding to their reported effect sizes and corresponding allele dosages. These scores were obtained by Bayesian method in pooled genome-wide association study (GWAS), which were previously described in detail (33). Latest GWAS of Low back pain including 119,216 cases and 132,641 controls of European ancestry was selected for computing PRS score (34). 174 genetic loci were reported to be related ti Low back pain (*p* < 5 × 10^−8^), and finally 8 SNPs were retrieved after removing the linkage disequilibrium. In addition, PRS was categorized as low (Q1), intermediate (Q2-Q3), and high (Q4) genetic risk.

### Covariates

We adjusted for a range of confounders. Through touchscreen questionnaires, baseline verbal interviews, physical measurements, and sample assays, participants provided information on age, sex, race, the Townsend Deprivation Index (TDI), education level, employment status, assessment centre, current smoking, drinking frequency, sleep duration, systolic blood pressure (SBP), physical activity, steroid medication use, and depression-related symptoms. Specifically, drinking status was classified as daily, 1–3 times per week, 1–3 times per month, only on special occasions, and never. Smoking status was classified as current smoking versus not. Depressive symptoms were measured at baseline using the 2-item Patient Health Questionnaire (PHQ-2), which assesses the frequency of depressed mood and anhedonia over the preceding 2 weeks. Each item is scored from 0 to 3, yielding a total score ranging from 0 to 6. A score of ≥3 is commonly used to indicate possible depressive disorder (35, 36). Physical activity was assessed at baseline using a modified version of the short-form International Physical Activity Questionnaire (IPAQ). Total physical activity was calculated as the sum of walking, moderate-intensity, and vigorous-intensity activities performed in the past week and expressed as metabolic equivalent of task (MET)-minutes per week (37). Baseline steroid medication use was assessed using field 20,003, which records the codes for medications or treatments regularly taken by participants, self-reported through a structured verbal interview at the assessment centre. Steroid medications included hydrocortisone, betamethasone and its esters, prednisone, dexamethasone, budesonide, fluticasone, mometasone, clobetasol, and triamcinolone, among others, corresponding to 224 specific codes listed in Supplementary Table 2.

We also adjusted for coronary heart disease, stroke, type 2 diabetes mellitus (T2DM), and osteoarthritis. The ICD codes related to coronary heart disease, stroke, and osteoarthritis are listed in Supplementary Table 1. T2DM was defined as meeting one or more of the following criteria: a diabetes diagnosis recorded by International Classification of Diseases, Ninth (ICD-9) or Tenth (ICD-10) Revision codes; a physician-diagnosed history of diabetes; current use of antidiabetic medication; a glycated hemoglobin (HbA1c) level ≥6.5%; a fasting (8-h) plasma glucose (FPG) level ≥7.0 mmol/L; or a random plasma glucose (RPG) level ≥11.1 mmol/L (38). All codes used to define diabetes are listed in Supplementary Table 3. For variables with less than 10% missingness, missing values were imputed using multiple imputation by chained equations (MICE); the extent of missing data for each variable is presented in Supplementary Table 4.

### Statistical analysis

Baseline characteristics of participants were described separately according to back pain status. Categorical variables were expressed as percentages (%), normally distributed continuous variables as mean ± standard deviation (SD), and non-normally distributed continuous variables as median and interquartile range (IQR). Between-group differences were assessed using Student’s t-test for normally distributed continuous variables and the Mann–Whitney U test for non-normally distributed continuous variables. Categorical variables were compared using the chi-square (χ^2^) test or Fisher’s exact test.

We used multivariable Cox proportional hazards regression models to assess the associations of the adiposity indices and the TyG index with the risk of incident back pain, with effect estimates expressed as hazard ratios (HRs) and 95% confidence intervals (CIs). Model 1 was adjusted for age, sex, race, education level, employment status, TDI, and assessment centre. Model 2 was further adjusted, on top of Model 1, for current smoking status, drinking status, sleep duration, SBP, depressive symptoms, CHD, stroke, diabetes, steroid use, and osteoarthritis. HRs and 95% CIs were estimated per 1-SD increment of each continuous adiposity index and across categories of each index, with the lowest category as the reference. In addition, we examined the dose–response relationship between the adiposity indices and the risk of incident back pain using restricted cubic splines (RCS) nested within the Cox models. Finally, model discrimination was assessed using Harrell’s C-index. We fitted separate Cox models, each including one of the six adiposity indices (with the same covariates as Model 2), and computed the C-index and its 95% confidence interval for each. Using the BMI model as the reference, differences in the C-index between each of the other five indices and the BMI model were compared using a z-test. To evaluate the robustness of our results, we performed the following sensitivity analyses. First, we further adjusted for physical activity duration in the subset of participants with physical activity duration data (*n* = 294,961). Second, we re-assessed the associations of the adiposity indices and the TyG index with the risk of incident back pain in the complete-covariate cohort (n = 341,588). Third, we assessed the proportional hazards assumption using Schoenfeld residuals. Because the main exposures showed evidence of non-proportional hazards, we further fitted piecewise time-varying-effect Cox models, dividing the follow-up period into 0–5, 5–10, and >10 years and estimating interval-specific HRs and 95% CIs. A likelihood ratio test was used to compare the constant-effect model with the piecewise time-varying-effect model, in order to test whether the effects of the main exposures varied over follow-up.

When analyzing mediating effects, the TyG index was treated as the mediator, and two models were constructed: a mediator model (GLM) to examine the association between obesity index and the TyG index (mediator); and a Cox regression including both the exposure and the mediator. The covariates were the same as in Model 2 except for the exclusion of diabetes. The proportion mediated was determined, and its significance was tested using bootstrap resampling.

To investigate the correlation in populations with different genetic risks for PRS, stratified analyses were performed with individuals with a low obesity index as the reference group. Meanwhile, in order to analyze the combined effect of PRS and obesity on back pain risk, patients have been further divided into 9 subgroups by PRS (low, medium, and high) and obesity index (low, medium, high). We also estimated the HR of back pain in different groups compared to individuals with low PRS and minimal obesity index. In order to quantify the interaction of summation and multiplication, we additionally included the interaction term of the obesity index and PRS in our model. Relative excess risk due to interaction (RERI) and 95% CI were calculated using approximate variance estimates.

## Results

### Baseline characteristics of participants

With an average follow-up duration of 14.37 years, 29,821 (7.9%) of 379,646 participants developed new-onset back pain; baseline characteristics are shown in Table 1. The cohort had a mean age of 56.5 years, with 53.4% female participants and 94.8% identifying as White. Compared with those without back pain, individuals who developed back pain were more frequently female, more highly educated, more likely to be unemployed, and more socioeconomically deprived (higher Townsend index). They also smoked more often and drank less frequently, and were more likely to have depressive symptoms, diabetes, CHD, stroke, and osteoarthritis, as well as higher levels of all adiposity indices.

**Table 1:** Baseline characteristics from the UK Biobank, by back pain status.

Variables	Overall (*n* = 379,646)	No back pain (*n* = 349,825)	Back pain (*n* = 29,821)	*p*-value
Age, y	56.52 (8.11)	56.42 (8.11)	57.71 (8.03)	<0.001
Sex				<0.001
Male	176,985 (46.62)	164,233 (46.95)	12,752 (42.76)	
Female	202,661 (53.38)	185,592 (53.05)	17,069 (57.24)	
Race				<0.001
White	359,794 (94.77)	331,804 (94.85)	27,990 (93.86)	
Non-White	19,852 (5.23)	18,021 (5.15)	1831 (6.14)	
Townsend index (TDI)	17.14 (13.94)	16.98 (13.82)	19.10 (15.18)	<0.001
Education				<0.001
College or University degree	64,305 (16.94)	57,184 (16.35)	7,121 (23.88)	
A, O levels and CSEs or equivalent	44,909 (11.83)	40,874 (11.68)	4,035 (13.53)	
NVQ\HND\HNC\Other professional qualifications	144,582 (38.08)	133,561 (38.18)	11,021 (36.96)	
None of the above	125,850 (33.15)	118,206 (33.79)	7,644 (25.63)	
Unemployment	28,667 (7.55)	25,546 (7.30)	3,121 (10.47)	<0.001
SBP, mmHg	141.72 (19.46)	141.68 (19.46)	142.19 (19.48)	<0.001
Drink status				<0.001
Daily	78,480 (20.67)	72,829 (20.82)	5,651 (18.95)	
1-4/w	187,054 (49.27)	173,651 (49.64)	13,403 (44.94)	
1-3/y, special	84,628 (22.29)	77,093 (22.04)	7,535 (25.27)	
Never	29,484 (7.77)	26,252 (7.50)	3,232 (10.84)	
Current smoke status	39,299 (10.35)	35,471 (10.14)	3,828 (12.84)	<0.001
Depressive status	21,872 (5.76)	19,272 (5.51)	2,600 (8.72)	<0.001
Coronary heart disease	18,585 (4.90)	16,228 (4.64)	2,357 (7.90)	<0.001
Stroke	6,911 (1.82)	6,062 (1.73)	849 (2.85)	<0.001
Diabetes	23,710 (6.25)	21,024 (6.01)	2,686 (9.01)	<0.001
Osteoarthritis	13,128 (3.46)	11,223 (3.21)	1905 (6.39)	<0.001
Height, cm	168.61 (9.27)	168.70 (9.26)	167.57 (9.36)	<0.001
TC, mg/dL	1.48 [1.04,2.14]	1.47 [1.04,2.13]	1.56 [1.10,2.25]	<0.001
Obesity index				
BMI > 30	91,405 (24.08)	82,067 (23.46)	9,338 (31.31)	<0.001
Waist circumference>102/88	126,203 (33.24)	113,767 (32.52)	12,436 (41.70)	<0.001
High hip circumference	98,914 (26.05)	89,388 (25.55)	9,526 (31.94)	<0.001
High WHR	95,012 (25.03)	86,729 (24.79)	8,283 (27.78)	<0.001
High ABSI	94,912 (25.00)	86,984 (24.87)	7,928 (26.59)	<0.001
WHtR >0.6	68,746 (18.11)	61,298 (17.52)	7,448 (24.98)	<0.001

BMI, Body mass index; WHR, Waist-hip Ratio; ABSI, A body shape index; WHtR, Waist-to-height ratio.

### Association of obesity index with back pain risk

**Figure 1:**
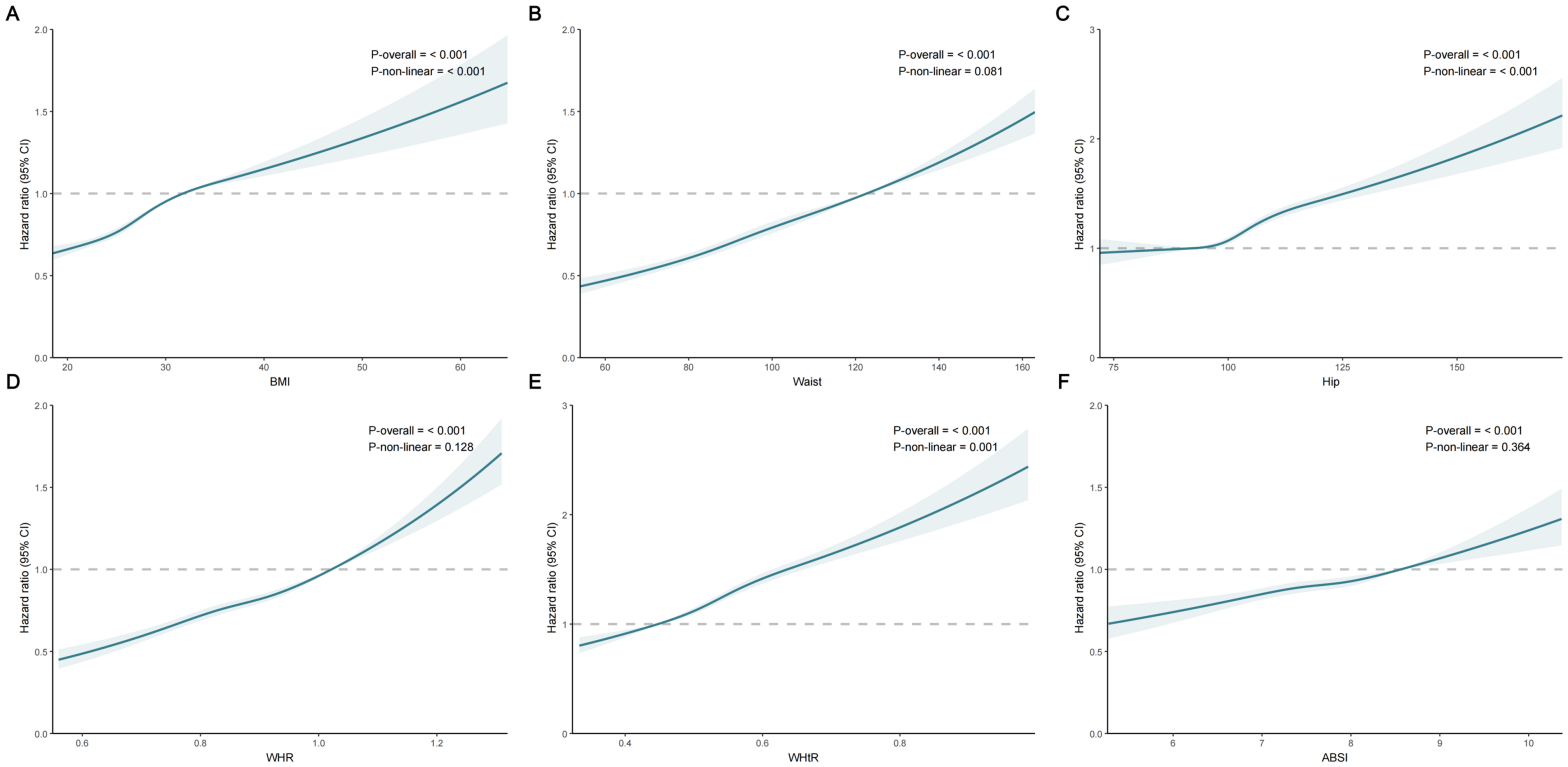
Dose–response association between obesity index and the incidence of back pain. **(A)** BMI; **(B)** Waist circumference; **(C)** Hip; **(D)** WHR; **(E)** WHtR; **(F)** ABSI. HR, hazard ratio; CI, confidence interval; BMI: Body mass index; WHR: Waist-hip ratio; ABSI: A body shape index; WHtR: Waist-to-height ratio. Models were adjusted for age, sex, race, education level, employment status, TDI, assessment centre, current smoking status, drinking status, sleep duration, SBP, depressive symptoms, CHD, stroke, diabetes, steroid use, and osteoarthritis.

Table 2 shows the findings of the Cox regression on the adiposity indices and the risk of back pain. Because some covariates violated the proportional hazards assumption, the results reflect the average hazard over the 14.37-year follow-up. In the multivariate analysis model, all categorical variables of obesity index were related to greater back pain risk. Specifically, in the fully adjusted model, each 1-SD increase in BMI, waist circumference, hip circumference, WHR, ABSI, and WHtR was associated with a 15% (95% CI: 1.13–1.16), 18% (1.16–1.19), 12% (1.11–1.14), 15% (1.13–1.17), 6% (1.04–1.07), and 16% (1.15–1.17) higher risk of incident back pain, respectively. High BMI, high Waist circumference, high hip circumference, high WHR high ABSI, and high WHtR were connected to a 45% (95% CI: 1.40 to 1.49), 38% (95% CI: 1.34 to 1.42), 36% (95% CI: 1.32 to 1.41), 30% (95% CI: 1.35 to 1.25), 14% (95% CI: 1.10 to 1.18), and 50% (95% CI: 1.45 to 1.55) greater back pain risk. Furthermore, results from RCS regression model were presented in Figure 1, which demonstrated the dose–response correlation of obesity index and back pain risk in fully adjusted model. Specifically, higher obesity index was connected to greater risk of back pain. Using RCS regression, BMI, hip circumference, and WHtR showed non-linear associations with back pain risk (all *p* for non-linearity < 0.05), whereas waist circumference, WHR, and ABSI were linearly associated with back pain risk (*p* for non-linearity = 0.081, 0.128, and 0.364, respectively).

**Table 2:** Association between obesity index and back pain risk.

Variables	Model 1		Model 2		Proportion mediated
	HR	*p*-value	HR	*p*-value	
TyG					
Per SD increment	1.09 (1·0.08–1.10)	<0.001	1.07 (1.05–1.08)	<0.001	**-**
Low	Reference		Reference		
Medium	1.09 (1.06–1.12)	<0.001	1.08 (1.05–1.11)	<0.001	**-**
High	1.24 (1.20–1.28)	<0.001	1.18 (1.14–1.22)	<0.001	**-**
BMI					
Per SD increment	1.17 (1.16–1.18)	<0.001	1.15 (1.13–1.16)	<0.001	7.6% (4.6 to 10.2%)
Low	Reference		Reference		
Medium	1.19 (1.16–1.22)	<0.001	1.19 (1.15–1.22)	<0.001	9.3% (6.2 to 14.1%)
High	1.51 (1.47–1.56)	<0.001	1.45 (1.40–1.49)	<0.001	8.9% (5.2 to 13.0%)
Waist circumference					
Per SD increment	1.21 (1.19–1.22)		1.18 (1.16–1.19)		6.7% (3.5 to 9.0%)
Low	Reference		Reference		
Medium	1.18 (1.15–1.22)	<0.001	1.18 (1.14–1.21)	<0.001	7.8% (3.7 to 12.5%)
High	1.44 (1.40–1.48)	<0.001	1.38 (1.34–1.42)	<0.001	10.2% (6.2 to 14.3%)
Hip circumference					
Per SD increment	1.14 (1.13–1.15)		1.12 (1.11–1.14)		9.1% (7.2 to 11.5%)
Low	Reference		Reference		
Medium	1.12 (1.09–1.16)	<0.001	1.13 (1.09–1.16)	<0.001	11.0% (6.0 to 15.6%)
High	1.40 (1.36–1.45)	<0.001	1.36 (1.32–1.41)	<0.001	10.6% (8.0 to 13.3%)
WHR					
Per SD increment	1.19 (1.17–1.21)		1.15 (1.13–1.17)		14.0% (9.4 to 17.7%)
Low	Reference		Reference		
Medium	1.18 (1.14–1.21)	<0.001	1.15 (1.11–1.18)	<0.001	17.9% (11.2 to 24.4%)
High	1.39 (1.34–1.44)	<0.001	1.30 (1.25–1.35)	<0.001	19.8% (14.4 to 27.2%)
WHtR					
Per SD increment	1.19 (1.18–1.20)		1.16 (1.15–1.17)		6.8% (3.5 to 9.9%)
Low	Reference		Reference		
Medium	1.22 (1.19–1.26)	<0.001	1.21 (1.18–1.25)	<0.001	9.6% (4.0 to 15.7%)
High	1.60 (1.54–1.65)	<0.001	1.50 (1.45–1.55)	<0.001	9.1% (3.2 to 14.2%)
ABSI					
Per SD increment	1.08 (1.07–1.10)	<0.001	1.06 (1.04–1.07)	<0.001	26.6% (20.3 to 37.2%)
Low	Reference		Reference		
Medium	1.11 (1.07–1.14)	<0.001	1.09 (1.06–1.12)	<0.001	35.1% (22.4 to 64.0%)
High	1.20 (1.16–1.25)	<0.001	1.14 (1.10–1.18)	<0.001	31.3% (22.1 to 46.5%)

BMI, Body mass index; WHR, Waist-hip Ratio; ABSI, A body shape index; WHtR, Waist-to-height ratio; TyG, Triglyceride-Glucose.

Supplementary Table 5 presents the results of the sensitivity analyses. First, in the subset of participants with physical activity (metabolic equivalent of task, MET) data (*n* = 294,961), the main results remained stable after further adjustment for physical activity level. Second, in the complete-case analysis among participants with complete covariate data (*n* = 339,797), the associations of the adiposity indices and the TyG index with the risk of incident back pain were not materially changed.

Third, the piecewise time-varying-effect Cox models (Supplementary Table 6) showed that the associations of each index with incident back pain varied in strength over follow-up, with statistically significant time-varying effects for all indices and the TyG index (P for temporal variation ≤ 0.05). Nevertheless, the direction of the associations remained consistent across all intervals, with most indices showing stronger associations at later follow-up [e.g., the HR per 1-SD increment in the TyG index rose from 1.03 (95% CI: 1.01–1.05) at 0–5 years to 1.11 (1.08–1.13) at >10 years]. Thus, despite the non-proportional hazards, the direction of the associations with incident back pain was consistent over time.

To compare the discriminatory performance of the six adiposity indices on a common scale, we computed Harrell’s C-index for each (Supplementary Table 7). The indices showed comparable discrimination for incident back pain, with C-indices clustering around 0.62 (range 0.614–0.620). Using the BMI model (C-index = 0.620) as the reference, none of the other indices differed significantly except ABSI, which had a slightly lower C-index (0.614, *p* = 0.0095); the differences for waist circumference, hip circumference, WHR, and WHtR were not statistically significant (*p* = 0.964, 0.394, 0.193, and 0.914, respectively).

### Interaction as well as joint analysis of obesity index and genetic risk with back pain events

**Figure 2:**
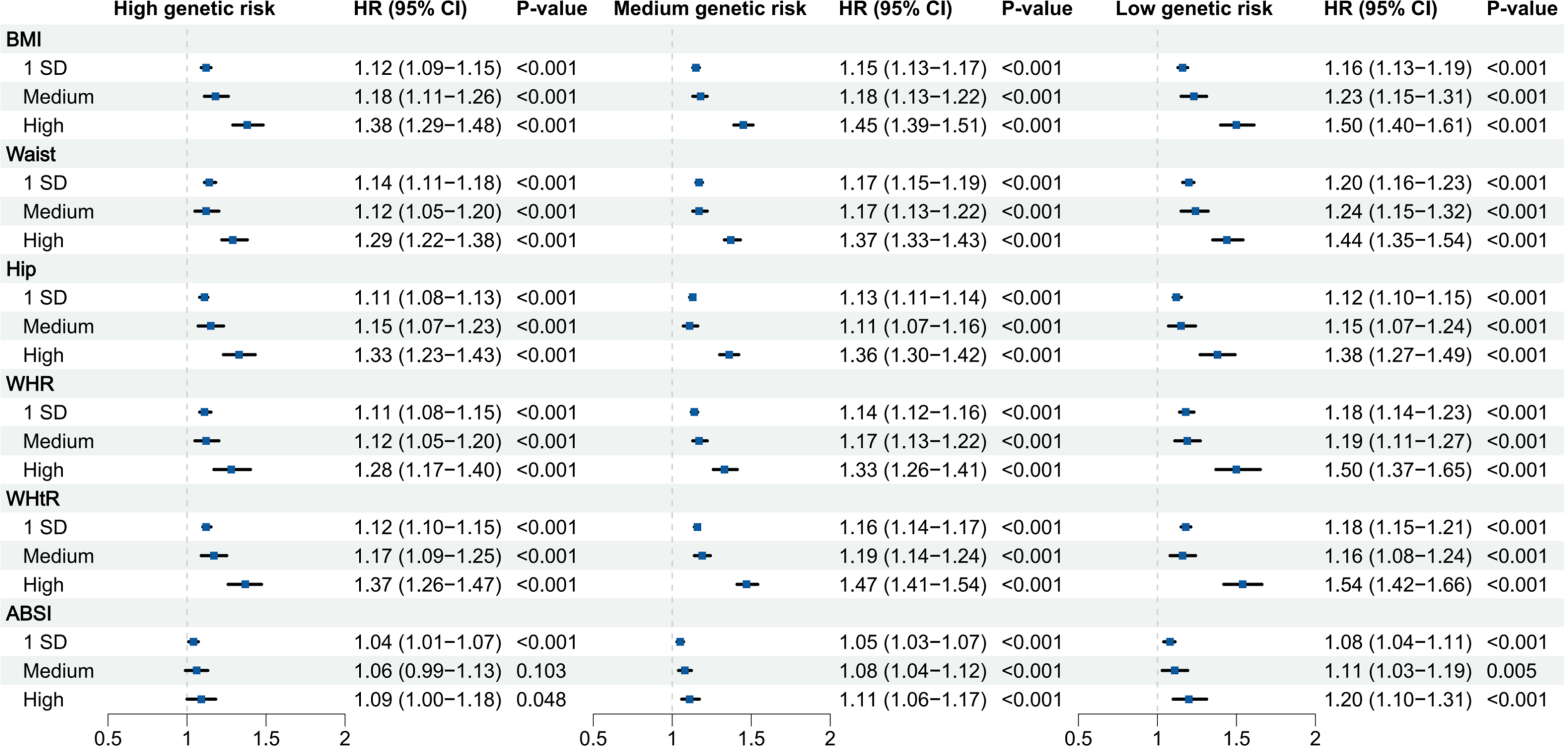
Associations of BMI and back pain incidence risk by PRS. CI, confidence interval; BMI: Body mass index; WHR: Waist-hip ratio; ABSI: A body shape index; WHtR: Waist-to-height ratio. Models were adjusted for age, sex, race, education level, employment status, TDI, assessment centre, current smoking status, drinking status, sleep duration, SBP, depressive symptoms, CHD, stroke, diabetes, steroid use, and osteoarthritis.

Higher adiposity indices were associated with higher back pain risk across all genetic risk subgroups, with associations generally stronger in the low genetic risk group. As shown in Figure 2, after full adjustment, among participants with low genetic risk, a high category of BMI, waist circumference, hip circumference, WHR, WHtR, and ABSI was associated with a 50% (95% CI: 1.40–1.61), 44% (1.35–1.54), 38% (1.27–1.49), 50% (1.37–1.65), 54% (1.42–1.66), and 20% (1.10–1.31) higher risk of back pain, respectively. Comparable associations were observed in the medium genetic risk group (HRs 1.45, 1.37, 1.36, 1.33, 1.47, and 1.11, respectively) and the high genetic risk group (HRs 1.38, 1.29, 1.33, 1.28, 1.37, and 1.09, respectively), indicating that the adiposity–back pain associations persisted regardless of genetic risk.

**Figure 3:**
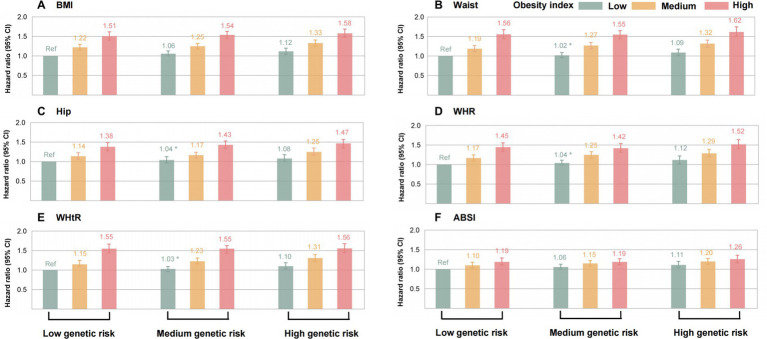
Joint associations of BMI and PRS with back pain incident risk **(A)**, waist circumference **(B)**, hip **(C)**, WHR **(D)**, WHtR **(E)**, and ABSI **(F)**. CI, confidence interval; BMI, Body mass index; WHR, Waist-hip ratio; ABSI, A body shape index; WHtR, Waist-to-height ratio. Models were adjusted for age, sex, race, education level, employment status, TDI, assessment centre, current smoking status, drinking status, sleep duration, SBP, depressive symptoms, CHD, stroke, diabetes, steroid use, and osteoarthritis.

In addition, the joint effect of obesity index and genetic susceptibility on back pain risk could be found on Figure 3. The group with high obesity index and high genetic risk was related to a significantly elevated risk. Specifically, high BMI (HR: 1.58; 95%CI: 1.48,1.69; *p* < 0.001), high waist circumference (HR = 1.62; 95%CI: 1.51–1.75; *p* < 0.001), high hip circumference (HR = 1.47; 95%CI: 1.35–1.51; *p* < 0.001), high WHR (HR = 1.52; 95%CI: 1.41–1.64; p < 0.001), high WHtR (HR = 1.56; 95%CI: 1.45–1.68; *p* < 0.001) and high ABSI (HR = 1.26; 95%CI: 1.17–1.36; *p* < 0.001),. No significant multiplicative or additive interactions were found between all obesity indices and genetic susceptibility in our investigation.

### Mediation analysis of the effect of obesity index on incident back pain via TyG

**Figure 4:**
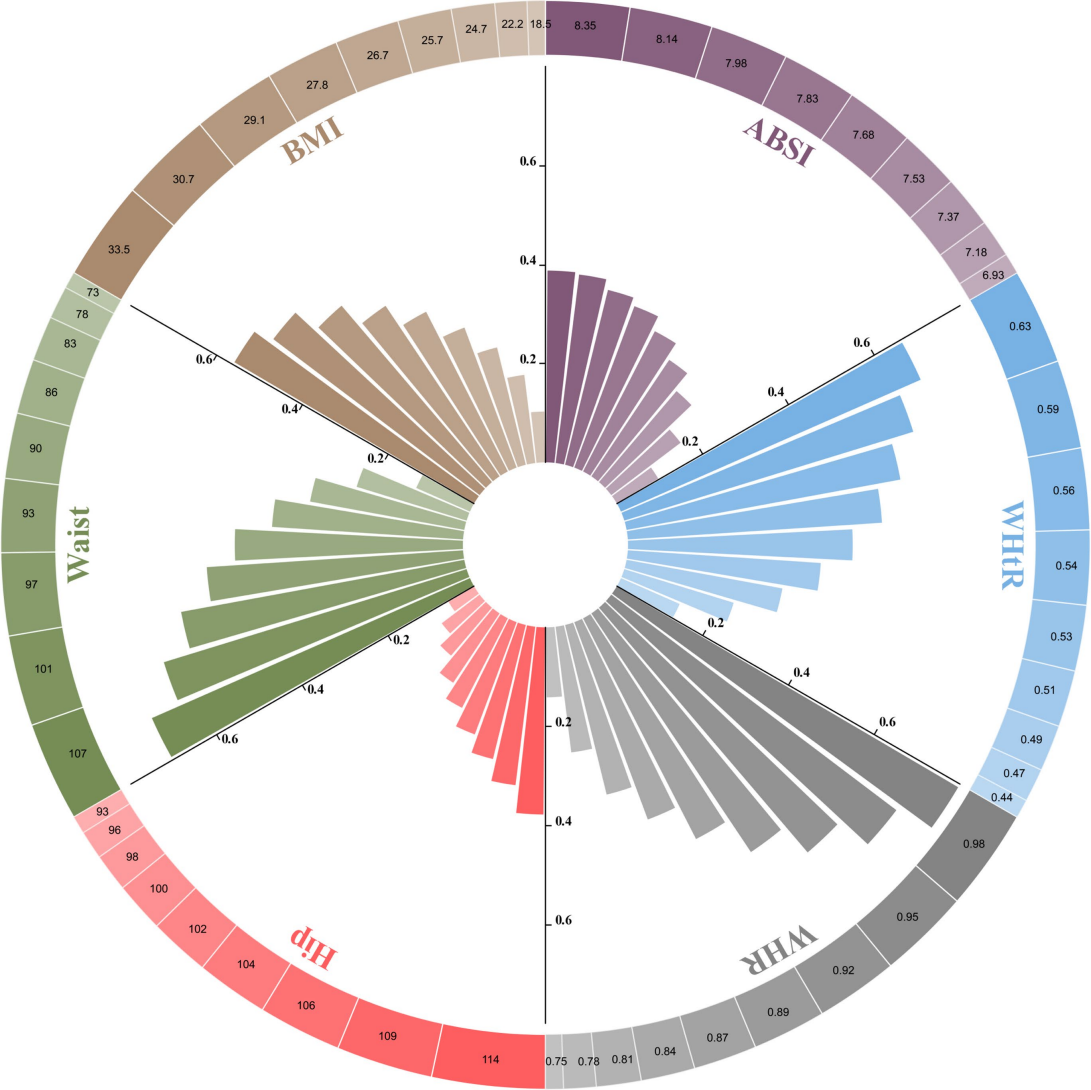
Associations of obesity indexes and TyG; BMI: Body mass index; WHR, Waist-hip ratio; ABSI, A body shape index; WHtR, Waist-to-height ratio. Models were adjusted for age, sex, race, education level, employment status, TDI, assessment centre, current smoking status, drinking status, sleep duration, SBP, depressive symptoms, CHD, stroke, diabetes, steroid use, and osteoarthritis.

After full adjustment, high TyG was connected to 18% (95% CI: 1.14 to 1.22) higher risk of back pain (Table 2). Also, as shown in Figure 4, higher obesity index was associated with higher TyG. Specifically, the top 10% of BMI, waist circumference, hip circumference, WHR, ABSI, and WHtR were linked to TyG elevations ranging from 0.37 to 0.80 units. Also, mediation analyses showed that TyG index mediated the interaction among different obesity indices and back pain in the fully adjusted model. Specifically, the proportions mediated by TyG for high BMI, high waist circumference, high hip circumference, high WHR, high WHtR and high ABSI, were 8.9% (5.2 to 13.0%), 10.2% (6.2 to 14.3%), 10.6% (8.0 to 13.3%), 19.8% (14.4 to 27.2%), 9.1% (3.2 to 14.2%), and 31.3% (22.1 to 46.5%), respectively.

## Discussion

Based on a prospective cohort of 379,646 UK Biobank participants, we examined the associations of six adiposity indices with incident back pain, as well as the mediating role of the TyG index and the joint associations of adiposity and genetic susceptibility. Higher adiposity indices were consistently associated with a higher risk of back pain, and the TyG index partially mediated these associations. The associations were present across all genetic risk strata, with no significant interaction between adiposity and genetic susceptibility, suggesting that adiposity may be associated with back pain largely independently of genetic predisposition.

A key strength of our study is the simultaneous comparison of six adiposity indices on a common scale, which addresses a notable gap in the existing literature. For instance, an analysis of 6,868 participants across three waves of the British birth cohort found that a 10% reduction in BMI was associated with a lower risk of low back pain (95% CI: 0.73–0.92) (39). Similarly, another study of 4,793 participants in the Osteoarthritis Initiative reported that higher BMI was associated with a greater risk of low back pain (95% CI: 1.010–1.046) (10). However, these studies relied predominantly on BMI, which reflects only overall weight relative to height and cannot capture regional fat distribution (11). In contrast, waist circumference, hip circumference, WHR, ABSI and WHtR reflected fat distribution more accurately. Waist circumference directly reflected abdominal fat accumulation, while Hip circumference represented the total subcutaneous fat, gluteus and hamstring muscles in the buttocks. WHR was the combination of the two, which better reflected centripetal obesity. Moreover, the WHtR further considered the effect of height on waist circumference. Finally, ABSI integrated waist circumference, BMI, and height, which can more accurately evaluate visceral fat accumulation. Unfortunately, few scholars have explored the interaction of obesity indices other than BMI and back pain risk. A study of 14,268 individuals reported that higher ABSI was associated with greater back pain risk (*p* = 0.008) (40). Furthermore, a study of 2,575 participants from Finnish Young Cardiovascular Risk Study indicated that a waist circumference greater than 88 cm was connected to 80% more back pain risk (95% CI: 1.0–3.2) (9). These earlier studies were nonetheless limited by cross-sectional designs or modest sample sizes, constraining causal interpretation.

Beyond confirming the association, our standardized analyses offer a more nuanced interpretation of how these indices compare. On a common per-SD scale, the effect sizes were broadly similar across indices: each 1-SD increment in BMI, waist circumference, hip circumference, WHR, ABSI, and WHtR was associated with a 15, 18, 12, 15, 6, and 16% higher risk of back pain, respectively. Consistent with this, the C-indices of all six indices clustered around 0.62, with no significant differences from BMI except for ABSI (p = 0.009). This indicates that the apparently large differences seen with categorical HRs (e.g., BMI vs. ABSI) were partly driven by differing categorization schemes rather than by true differences in discriminatory performance—an important caveat for interpreting and comparing adiposity indices in this field. We further observed nonlinear associations for BMI and hip circumference and approximately linear associations for waist circumference, WHR, ABSI, and WHtR, though the modest discriminatory ability suggests that no single anthropometric index strongly predicts back pain at the individual level.

The effects of obesity on back pain were complicated. Mechanically, greater adiposity increases spinal loading, and sustained loading may contribute to structural changes in the spine, such as intervertebral disc degeneration and reduced disc height, that are associated with back pain (41). Biologically, adipose tissue can influence bone and disc metabolism through endocrine and paracrine mediators, including adipokines such as leptin and adiponectin and pro-inflammatory cytokines such as TNF-*α* and IL-6 (42). White adipocytes secreted not only hormones including lipocalin and leptin, but also pro-inflammatory factors, which negatively regulated bone metabolism (43). Therefore, there should be studies to comprehensively reveal the association between obesity and back pain risk. Here we figured out the correlation of multiple obesity indicators and back pain risk, with results showing a significant correlation, which was in line with prior works (10, 40, 44). Finally, nonlinear (BMI, hip circumference) and linear (waist circumference, WHR, ABSI and WHtR) correlation were found with back pain risk.

Genetic susceptibility has also been associated with back pain risk (21). To examine the joint associations of obesity and genetic risk, we constructed a PRS for back pain, but observed no significant multiplicative or additive interaction. Across low, medium, and high genetic risk strata, higher adiposity was consistently associated with back pain, and the associations were of similar or even greater magnitude in the low genetic risk group (e.g., for high WHtR, HRs of 1.54, 1.47, and 1.37 in the low, medium, and high genetic risk groups, respectively). This pattern suggests that the adiposity–back pain association is unlikely to be substantially modified by genetic predisposition, and that adiposity may be associated with back pain across the spectrum of genetic risk. However, given that this PRS was derived largely from low back pain GWAS, its relevance to our broader outcome should be interpreted cautiously.

Finally, our findings highlight a potential metabolic pathway linking adiposity to back pain. Obesity is associated with insulin resistance and oxidative stress (14–16), which may in turn relate to sarcopenic obesity (45) and a greater mechanical and metabolic burden on the spine. Obesity is also associated with metabolic syndrome, osteoarthritis, and osteoporosis, conditions that may further relate to back pain. To our knowledge, this is among the first studies to examine the TyG index—a surrogate marker of insulin resistance—as a mediator of the adiposity–back pain association. The TyG index partially mediated these associations, with proportions mediated of 8.9, 10.2, 10.6, 19.8, 9.1, and 31.3% for high BMI, waist circumference, hip circumference, WHR, WHtR, and ABSI, respectively. The comparatively larger mediation proportions for WHR and ABSI suggest that insulin resistance may be particularly relevant to the link between central or visceral adiposity and back pain. These findings point to insulin resistance and metabolic dysfunction as factors worth attention in individuals with obesity; however, given the exploratory and cross-sectional nature of the mediation analysis, whether targeting these factors reduces back pain risk remains to be tested in future studies.

There were some main strengths in our work. First, we based the large cohort with extended follow-up duration provided by UK Biobank. The adequate sample size allowed us to conduct joint and stratified analyses with sufficient statistical power, offering more robust conclusions. In addition, we generated back pain PRS scores to assess the joint relationship between genetic susceptibility and obesity. However, limitations were inevitable. First, the UK Biobank database recruited predominantly people aged 40 years or older in the UK, so careful consideration was needed when generalizing the results of this study. Second, we did not collect the long-term obesity index profile of participants, leaving the possibility of obesity changes. Third, there might be reverse causality in prospective cohort studies between obesity and back pain. Fourth, the back pain outcome in this study was based on the UK Biobank “First Occurrence” fields, which are mapped to the 3-character ICD-10 code M54, encompassing different anatomical regions such as cervical, thoracic, and low back pain, and could not be disaggregated into specific subcodes owing to this coding granularity. Our outcome therefore reflects back pain in its broad sense rather than low back pain specifically. As prior evidence has largely focused on low back pain, the extrapolation of its mechanistic interpretations to the broad back pain outcome in this study warrants caution; future studies with finer-grained coding could further examine region-specific associations. Fifth, occupational information was available only as employment status, without details on occupation type or the intensity of manual labor, so we could not precisely capture occupational mechanical loading. Sixth, the adiposity indices and the mediator (TyG index) were measured concurrently at baseline, which does not satisfy the temporal ordering required for a strict mediation framework. The mediation results should therefore be interpreted as an exploration of association pathways and effect decomposition rather than causal mediation, and reverse causation between the exposure and the mediator cannot be excluded. Seventh, this study was exploratory and hypothesis-generating in nature; the observed associations do not establish causality and should be interpreted with caution and confirmed in future studies. Finally, although we adjusted for a broad set of covariates, there could still be residual confounders that precluded causal inferences.

## Conclusion

Collectively, using a large prospective cohort, we found that higher adiposity indices were associated with an increased risk of back pain, with the insulin resistance surrogate TyG index partially mediating these associations. No significant interaction was observed between adiposity and genetic susceptibility, suggesting that the association may be largely independent of genetic predisposition. These findings highlight insulin resistance and metabolic dysfunction as factors worth attention in individuals with obesity; however, given the exploratory nature of this study, whether addressing them reduces the burden of back pain warrants confirmation in future research.

## Data Availability

Publicly available datasets were analyzed in this study. This data can be found at: https://www.ukbiobank.ac.uk.
