# Supplementary material for: Associations of six adiposity indices with incident back pain and the mediating role of the triglyceride-glucose index: prospective findings from the UK biobank
Source: Front Nutr. 2026 Aug 5;13:1837601. doi: 10.3389/fnut.2026.1837601 (PMC13485806; doi:10.3389/fnut.2026.1837601)
Supplement: Supplementary file 1 [file Table_1.docx]

**Associations of Six Adiposity Indices with Incident Back Pain and the Mediating Role of the Triglyceride-Glucose Index: Prospective Findings from the UK Biobank**

[**Figure S1.** Flowchart of participant selection and cohort construction in the prospective cohort study.	2](#_Toc233077069)

[**Table S1.** Codes used in the UK Biobank to identify Disease.	3](#_Toc233077070)

[**Table S2.** UK Biobank medication codes used to define steroid use.	3](#_Toc233077071)

[**Table S3.** Codes used in the UK Biobank to identify T2DM.	11](#_Toc233077072)

[**Table S4.** The proportion of missing variables in the models.	12](#_Toc233077073)

[**Table S5.** Sensitivity analyses of the association between obesity index and incident back pain.	12](#_Toc233077074)

[**Table S6.** Time-varying associations of obesity and TyG indices with incident back pain	14](#_Toc233077075)

[**Table S7.** Harrell’s C-index among six adiposity indices for incident back pain.	15](#_Toc233077076)

**Figure S1.** Flowchart of participant selection and cohort construction in the prospective cohort study.

**Table S1.** Codes used in the UK Biobank to identify Disease.

| **Code** | **Diseases** |
| --- | --- |
| **Back pain** | |
| M54 | Dorsalgia |
| M54.0 | Panniculitis affecting regions of neck and back |
| M54.1 | Radiculopathy |
| M54.2 | Cervicalgia |
| M54.3 | Sciatica |
| M54.4 | Lumbago with sciatica |
| M54.5 | Low back pain |
| M54.6 | Pain in thoracic spine |
| M54.8 | Other dorsalgia |
| M54.9 | Dorsalgia, unspecified |
| **Coronary Artery Disease** | |
| I21 | Acute myocardial infarction |
| I22 | Subsequent myocardial infarction |
| I23 | Certain current complications following acute myocardial infarction |
| I24 | Other acute ischaemic heart diseases |
| I25 | Chronic ischaemic heart disease |
| **Stroke** |  |
| I60 | Subarachnoid haemorrhage |
| I61 | Intracerebral haemorrhage |
| I62 | Other nontraumatic intracranial haemorrhage |
| I63 | Cerebral infarction |
| I64 | Stroke, not specified as haemorrhage or infarction |
| **Arthritis** |  |
| M00 | Pyogenic arthritis |
| M05 | Seropositive rheumatoid arthritis |
| M06 | Other rheumatoid arthritis |
| M13 | Other arthritis |

**Table S2.** UK Biobank medication codes used to define steroid use.

|  | **Coding** | **Drug name** |
| --- | --- | --- |
| 1 | 1140851278 | betnovate compound suppository |
| 2 | 1140853448 | eumovate 0.1% eye drops 5ml |
| 3 | 1140853488 | neo-cortef eye drops |
| 4 | 1140853854 | gentamicin+hydrocortisone acetate 0.3%/1% ear drops |
| 5 | 1140853862 | neo-cortef ear drops 5ml |
| 6 | 1140854688 | desonide |
| 7 | 1140854784 | temetex 0.1% cream |
| 8 | 1140854788 | temetex 0.1% ointment |
| 9 | 1140854816 | fluocinonide |
| 10 | 1140854834 | fluocortolone |
| 11 | 1140854990 | halcinonide |
| 12 | 1140862380 | becloforte 250micrograms inhaler |
| 13 | 1140862382 | becotide 50 inhaler |
| 14 | 1140862474 | aerobec 50mcg autohaler |
| 15 | 1140862476 | beclazone 50 inhaler |
| 16 | 1140862572 | budesonide |
| 17 | 1140862574 | pulmicort ls 50micrograms inhaler |
| 18 | 1140862584 | pulmicort ls 50micrograms spacer inhaler |
| 19 | 1140864286 | flixotide 25micrograms inhaler |
| 20 | 1140864820 | cutivate 0.05% cream |
| 21 | 1140865838 | predsol 5mg suppository |
| 22 | 1140865840 | predfoam 20mg enema |
| 23 | 1140865922 | perinal spray |
| 24 | 1140868364 | prednisone |
| 25 | 1140868370 | decortisyl 5mg tablet |
| 26 | 1140868426 | triamcinolone |
| 27 | 1140868434 | ledercort 2mg tablet |
| 28 | 1140874784 | florinef 100mcg tablet |
| 29 | 1140874790 | betamethasone |
| 30 | 1140874794 | betnesol 500mcg soluble tablet |
| 31 | 1140874810 | cortistab 5mg tablet |
| 32 | 1140874814 | cortisyl 25mg tablet |
| 33 | 1140874816 | dexamethasone |
| 34 | 1140874822 | decadron 500micrograms tablet |
| 35 | 1140874896 | hydrocortisone |
| 36 | 1140874930 | prednisolone |
| 37 | 1140874936 | deltacortril enteric 2.5mg e/c tablet |
| 38 | 1140874940 | deltastab 1mg tablet |
| 39 | 1140874944 | precortisyl 1mg tablet |
| 40 | 1140874950 | prednesol 5mg tablet |
| 41 | 1140874954 | hydrocortistab 20mg tablet |
| 42 | 1140874956 | hydrocortone 10mg tablet |
| 43 | 1140874976 | methylprednisolone |
| 44 | 1140874978 | medrone 2mg tablet |
| 45 | 1140875668 | chloromycetin hydrocortisone eye ointment |
| 46 | 1140875684 | minims prednisolone 0.5% single-use eye drops |
| 47 | 1140875766 | fluorometholone |
| 48 | 1140875768 | fluorometholone+neomycin sulphate 0.1/0.5% eye drops |
| 49 | 1140875974 | locorten-vioform ear drops |
| 50 | 1140876036 | neomycin+hydrocortisone 0.5%/1.5% ear drops |
| 51 | 1140876044 | neomycin+hydrocortisone 0.5%/1.5% eye/ear ointment |
| 52 | 1140876046 | neomycin+hydrocortisone 0.5%/1.5% eye/ear drops |
| 53 | 1140876076 | flixonase 50micrograms aqueous nasal spray |
| 54 | 1140876104 | tri-adcortyl otic ear ointment |
| 55 | 1140876136 | beconase 50micrograms nasal spray |
| 56 | 1140876146 | rhinocort 50micrograms nasal spray |
| 57 | 1140876154 | flunisolide |
| 58 | 1140876240 | adcortyl in orabase oral paste |
| 59 | 1140876242 | corlan lozenge |
| 60 | 1140876456 | kerfoot hydrocortisone cream |
| 61 | 1140878052 | fml eye drops |
| 62 | 1140878056 | fml-neo eye drops |
| 63 | 1140878418 | alphosyl hc cream |
| 64 | 1140878562 | quinoderm+hydrocortisone cream |
| 65 | 1140879922 | coal tar solution+hydrocortisone 3%/0.25% cream |
| 66 | 1140879934 | coal tar extract+hydrocortisone 3%/0.25% cream |
| 67 | 1140881440 | anugesic-hc cream |
| 68 | 1140881442 | anusol hc ointment |
| 69 | 1140881444 | proctofoam hc foam |
| 70 | 1140881446 | proctosedyl ointment |
| 71 | 1140881448 | scheriproct ointment |
| 72 | 1140881454 | xyloproct ointment |
| 73 | 1140881922 | becodisks 100micrograms disks+diskhaler |
| 74 | 1140881938 | beclomethasone dipropionate+salbutamol |
| 75 | 1140882150 | propaderm ointment |
| 76 | 1140882152 | budesonide 0.025% cream |
| 77 | 1140882618 | diprosalic ointment |
| 78 | 1140882622 | betamethasone+salicylic acid |
| 79 | 1140882626 | betnesol 0.1% eye/ear/nose drops |
| 80 | 1140882630 | diprosone cream |
| 81 | 1140882694 | betnovate cream |
| 82 | 1140882708 | clobetasone butyrate+neomycin sulphate |
| 83 | 1140882718 | maxitrol eye drops |
| 84 | 1140882722 | maxidex eye drops |
| 85 | 1140882724 | sofradex eye/ear drops |
| 86 | 1140882730 | colifoam 10% foam |
| 87 | 1140882732 | dioderm cream |
| 88 | 1140882740 | mildison lipocream 1% cream |
| 89 | 1140882742 | efcortelan 0.5% cream |
| 90 | 1140882756 | timodine cream |
| 91 | 1140882764 | betamethasone+clioquinol |
| 92 | 1140882766 | betamethasone+neomycin sulphate |
| 93 | 1140882774 | betamethasone+fusidic acid |
| 94 | 1140882776 | fucibet cream |
| 95 | 1140882778 | lotriderm cream |
| 96 | 1140882780 | betamethasone+clotrimazole |
| 97 | 1140882782 | dermovate cream |
| 98 | 1140882794 | clobetasol propionate+neomycin sulphate+nystatin |
| 99 | 1140882800 | eumovate cream |
| 100 | 1140882806 | clobetasone butyrate+oxytetracycline+nystatin |
| 101 | 1140882818 | alphaderm cream |
| 102 | 1140882820 | calmurid hc cream |
| 103 | 1140882822 | hydrocortisone+urea |
| 104 | 1140882824 | hydrocortisone+pramoxine |
| 105 | 1140882830 | hydrocortisone+crotamiton |
| 106 | 1140882832 | otosporin ear drops |
| 107 | 1140882836 | vioform hydrocortisone cream |
| 108 | 1140882840 | hydrocortisone+sodium fusidate |
| 109 | 1140882842 | hydrocortisone+gentamicin |
| 110 | 1140882844 | hydrocortisone+nystatin |
| 111 | 1140882846 | hydrocortisone+potassium hydroxyquinoline |
| 112 | 1140882848 | hydrocortisone+oxytetracycline |
| 113 | 1140882850 | hydrocortisone+clioquinol |
| 114 | 1140882852 | locoid 0.1% cream |
| 115 | 1140882864 | hydrocortisone+chlorquinaldol |
| 116 | 1140882888 | hydrocortisone+chloramphenicol |
| 117 | 1140882892 | neo-cortef eye/ear ointment |
| 118 | 1140882894 | hydrocortisone+neomycin |
| 119 | 1140882896 | hydrocortisone+lignocaine |
| 120 | 1140882902 | hydrocortisone+dimethicone '350' |
| 121 | 1140882904 | canesten hc cream |
| 122 | 1140882906 | hydrocortisone+clotrimazole |
| 123 | 1140882908 | hydrocortisone+miconazole |
| 124 | 1140882910 | daktacort cream |
| 125 | 1140882914 | hydrocortisone+econazole |
| 126 | 1140882918 | hydrocortisone+fusidic acid |
| 127 | 1140882920 | fucidin h cream |
| 128 | 1140882926 | gentisone hc cream |
| 129 | 1140882928 | nystaform-hc 0.5% cream |
| 130 | 1140882934 | terra-cortril ointment |
| 131 | 1140882938 | terra-cortril nystatin cream |
| 132 | 1140883022 | neo-medrone 0.25% cream |
| 133 | 1140883026 | methylprednisolone+neomycin |
| 134 | 1140883028 | predsol 0.5% eye/ear drops |
| 135 | 1140883030 | pred forte 1% eye drops |
| 136 | 1140883034 | adcortyl+graneodin cream |
| 137 | 1140883038 | ledercort 0.1% cream |
| 138 | 1140883040 | adcortyl 0.1% cream |
| 139 | 1140883044 | tri-adcortyl cream |
| 140 | 1140883058 | triamcinolone+chlortetracycline |
| 141 | 1140883060 | triamcinolone+nystatin |
| 142 | 1140883062 | triamcinolone+econazole |
| 143 | 1140883064 | triamcinolone+neomycin |
| 144 | 1140884074 | gentisone hc ear drops |
| 145 | 1140884636 | diflucortolone |
| 146 | 1140884640 | nerisone 0.1% cream |
| 147 | 1140884642 | alclometasone |
| 148 | 1140884646 | modrasone 0.05% cream |
| 149 | 1140884654 | beclomethasone |
| 150 | 1140884660 | fluclorolone |
| 151 | 1140884672 | fludrocortisone |
| 152 | 1140884676 | fluocinolone |
| 153 | 1140884696 | clobetasone |
| 154 | 1140884700 | trimovate ointment |
| 155 | 1140884704 | cortisone product |
| 156 | 1140888074 | clobetasol |
| 157 | 1140888092 | elocon cream |
| 158 | 1140888098 | fluticasone |
| 159 | 1140888124 | uniroid ointment |
| 160 | 1140888134 | eczederm+hydrocortisone cream |
| 161 | 1140888142 | metosyn 0.05% cream |
| 162 | 1140888166 | ultralanum cream |
| 163 | 1140888168 | medrone lotion |
| 164 | 1140888172 | mometasone |
| 165 | 1140888176 | mometasone furoate 0.1% ointment |
| 166 | 1140888178 | haelan 0.0125% cream |
| 167 | 1140888184 | mometasone furoate 0.1% lotion |
| 168 | 1140888628 | hydrocortistab 1% cream |
| 169 | 1140888630 | hydrocortisyl 1% cream |
| 170 | 1140909786 | beclometasone |
| 171 | 1140909894 | desoximetasone |
| 172 | 1140910424 | hc - hydrocortisone |
| 173 | 1140910484 | cortisol product |
| 174 | 1140910634 | deltahydrocortisone |
| 175 | 1140910906 | eumovate eye drops |
| 176 | 1140913292 | synalar 1:10 cream |
| 177 | 1140916654 | neo-cortef ear drops 10ml |
| 178 | 1140916656 | neo-cortef eye ointment |
| 179 | 1140921024 | betacap scalp application |
| 180 | 1140926004 | entocort cr 3mg m/r capsule |
| 181 | 1140926988 | bettamousse 0.12% foam |
| 182 | 1141145782 | deflazacort |
| 183 | 1141146250 | entocort 2mg/100ml enema |
| 184 | 1141146508 | nasacort 55micrograms aqueous nasal spray |
| 185 | 1141150944 | nasonex 0.05% aqueous nasal spray |
| 186 | 1141151424 | hydrocortisone acetate+neomycin sulphate 1.5%/0.5% eye drops |
| 187 | 1141152074 | corlan pellet |
| 188 | 1141153270 | asmabec 50micrograms spacehaler |
| 189 | 1141157294 | hydrocortisone product |
| 190 | 1141157402 | prednisolone product |
| 191 | 1141157418 | budesonide product |
| 192 | 1141162532 | minims dexamethasone na phosphate 0.1% s-d eye drops |
| 193 | 1141163832 | budenofalk 3mg capsule |
| 194 | 1141164086 | salmeterol+fluticasone propionate |
| 195 | 1141166524 | rimexolone |
| 196 | 1141167174 | tramazoline hydrochloride+dexamethasone isonicotinate |
| 197 | 1141167594 | qvar 50 inhaler |
| 198 | 1141167708 | beclomist 50micrograms nasal spray |
| 199 | 1141169844 | dexamethasone+hypromellose 0.1%/0.5% eye drops |
| 200 | 1141173346 | cortisone |
| 201 | 1141174512 | budesonide+eformoterol |
| 202 | 1141174520 | symbicort 100/6 turbohaler |
| 203 | 1141174548 | dexamethasone+tobramycin |
| 204 | 1141174552 | tobradex eye drops |
| 205 | 1141176832 | seretide 50 evohaler |
| 206 | 1141176842 | dexsol 2mg/5ml oral solution |
| 207 | 1141179072 | pulvinal beclomethasone diprop 100mcg breath-act dry pdr inh |
| 208 | 1141179982 | betamethasone+calcipotriol |
| 209 | 1141180342 | beclometasone dipropionate+salbutamol |
| 210 | 1141181062 | hydrocortisone+dimeticone '350' |
| 211 | 1141181554 | clioquinol+flumetasone pivalate 1%/0.02% ear drops |
| 212 | 1141181610 | hydrocortisone+pramocaine |
| 213 | 1141182610 | fluocinolone acetonide+clioquinol 0.025%/3% cream |
| 214 | 1141182664 | fluocinolone acetonide+clioquinol 0.025%/3% ointment |
| 215 | 1141182666 | fluocinolone acetonide+neomycin sulphate 0.025%/0.5% cream |
| 216 | 1141182668 | fluocinolone acetonide+neomycin sulphate 0.025%/0.5% oint |
| 217 | 1141189464 | exe-cort hydrocortisone 1% cream |
| 218 | 1141191748 | respiratory mometasone |
| 219 | 1141192916 | rino clenil 50micrograms nasal spray |
| 220 | 1141194840 | hydrocortisone+lidocaine |
| 221 | 1141195232 | budesonide+formoterol |
| 222 | 1141195280 | novolizer budesonide 200micrograms/dose cartridge+inhaler |
| 223 | 1141200482 | fluprednidene |
| 224 | 1141200484 | fluprednidene + miconazole |

**Table S3.** Codes used in the UK Biobank to identify T2DM.

| **Diseases** | **Doctor-diagnosed / Medication / Operations / Laboratory biomarkers** | **ICD-9** | **ICD-10** |
| --- | --- | --- | --- |
| T2DM | 20003 (1140868902,1140874646, 1140874674, 1140874718, 1140874744, 1140883066, 1140884600, 1140921964, 1140874686, 1141152590, 1141157284, 1141168660,1141171646,1141173882,1141189090), 20002 (1220,1221,1223), 2443 (1), 6177 (3), 6153 (3), 30750 (HbA1c≥48 mmol/mol), 30740 (RPG≥11.1 mmol/L), 74 (8h-FPG≥ 7mmol/L) | 250,2500,25000,25009,2501,25010,25019,  2502,25020,25029,  2503,2504,2505,2506,  2507,2509, 25090, 25099 | E11 (E11\E11.0-E11.9) |
| T1DM | 20002 (1222) | 25001, 25011, 25021, 25091 | E10 (E10\E10.0-E10.9) |

**Abbreviations:** ICD-9, the International Classification of Diseases-Ninth Version; ICD-10, the International Classification of Diseases-Tenth Version; T2DM, type 2 diabetes mellitus; T1DM, type 1 diabetes mellitus.

**Table S4.** The proportion of missing variables in the models.

| **Variables** | **Proportion** |
| --- | --- |
|  |  |
| Education | 1.17% |
| Race | 0.47% |
| Townsend index | 2.52% |
| Career | 0.49% |
| Systolic pressure | 5.60% |
| Current smoking | 0.49% |
| Drink status | 0.21% |
| Physical activity (MET) | 22.3% |
| Sleep duration | 7.0% |
| Depressive symptoms | 6.73% |

**Table S5.** Sensitivity analyses of the association between obesity index and incident back pain.

|  | **Additional Physical activity Adjustment** | | **Complete-covariate cohort** | |
| --- | --- | --- | --- | --- |
|  | **Model 2** |  | **Model 2** |  |
|  | **HR** | **P-value** | **HR** | **P-value** |
| **TyG** |  |  |  |  |
| Per SD increment | 1.06 (1.04-1.07) | <0.001 | 1.07 (1.06-1.08) | <0.001 |
| Low | Reference |  | Reference |  |
| Medium | 1.08 (1.04-1.12) | <0.001 | 1.07 (1.04-1.11) | <0.001 |
| High | 1.16 (1.11-1.20) | <0.001 | 1.18 (1.14-1.23) | <0.001 |
| **BMI** |  |  |  |  |
| Per SD increment | 1.14 (1.12-1.15) | <0.001 | 1.14 (1.13-1.16) | <0.001 |
| Low | Reference |  | Reference |  |
| Medium | 1.20 (1.16-1.24) | <0.001 | 1.19 (1.15-1.23) | <0.001 |
| High | 1.44 (1.38-1.50) | <0.001 | 1.48 (1.42-1.53) | <0.001 |
| **Waist circumference** |  |  |  |  |
| Per SD increment | 1.16 (1.15-1.18) |  | 1.16 (1.15-1.18) |  |
| Low | Reference |  | Reference |  |
| Medium | 1.16 (1.12-1.20) | <0.001 | 1.17 (1.14-1.21) | <0.001 |
| High | 1.35 (1.31-1.39) | <0.001 | 1.38 (1.34-1.42) | <0.001 |
| **Hip circumference** |  |  |  |  |
| Per SD increment | 1.11 (1.10-1.13) |  | 1.11 (1.10-1.13) |  |
| Low | Reference |  | Reference |  |
| Medium | 1.12 (1.08-1.16) | <0.001 | 1.11 (1.08-1.15) | <0.001 |
| High | 1.34 (1.29-1.39) | <0.001 | 1.35 (1.30-1.40) | <0.001 |
| **WHR** |  |  |  |  |
| Per SD increment | 1.14 (1.12-1.16) |  | 1.14 (1.12-1.16) |  |
| Low | Reference |  | Reference |  |
| Medium | 1.16 (1.12-1.21) | <0.001 | 1.17 (1.13-1.21) | <0.001 |
| High | 1.36 (1.29-1.42) | <0.001 | 1.37 (1.31-1.41) | <0.001 |
| **WHtR** |  |  |  |  |
| Per SD increment | 1.15 (1.13-1.16) |  | 1.16 (1.14-1.17) |  |
| Low | Reference |  | Reference |  |
| Medium | 1.21 (1.18-1.25) | <0.001 | 1.22 (1.18-1.26) | <0.001 |
| High | 1.50 (1.45-1.55) | <0.001 | 1.50 (1.44-1.55) | <0.001 |
| **ABSI** |  |  |  |  |
| Per SD increment | 1.05 (1.04-1.07) | <0.001 | 1.06 (1.04-1.08) | <0.001 |
| Low | Reference |  | Reference |  |
| Medium | 1.09 (1.05-1.13) | <0.001 | 1.09 (1.05-1.13) | <0.001 |
| High | 1.14 (1.09-1.19) | <0.001 | 1.14 (1.09-1.18) | <0.001 |

BMI: body mass index; WHR: waist-hip Ratio; ABSI: a body shape index; WHtR: waist-to-height ratio; TyG: Triglyceride-Glucose.

**Table S6.** Time-varying associations of obesity and TyG indices with incident back pain

| **Exposure** | **Follow-up period** | **HR (95% CI, Model 2)** | **P value** | **P for temporal variation†** |
| --- | --- | --- | --- | --- |
| **TyG index, per SD** | Overall* | 1.07 (1.05-1.08) | <0.001 | <0.001 |
|  | 0–5 years | 1.03 (1.01-1.05) | <0.001 |  |
|  | 5–10 years | 1.08 (1.06-1.10) | <0.001 |  |
|  | >10 years | 1.11 (1.08-1.13) | <0.001 |  |
| **BMI, per SD** | Overall* | 1.15 (1.13-1.16) | <0.001 | 0.004 |
|  | 0–5 years | 1.13 (1.11-1.15) | <0.001 |  |
|  | 5–10 years | 1.15 (1.13-1.17) | <0.001 |  |
|  | >10 years | 1.18 (1.16-1.21) | <0.001 |  |
| **Waist, per SD** | Overall* | 1.18 (1.16-1.19) | <0.001 | <0.001 |
|  | 0–5 years | 1.14 (1.12-1.17) | <0.001 |  |
|  | 5–10 years | 1.17 (1.15-1.19) | <0.001 |  |
|  | >10 years | 1.24 (1.21-1.27) | <0.001 |  |
| **Hip, per SD** | Overall* | 1.12 (1.11-1.14) | <0.001 | 0.002 |
|  | 0–5 years | 1.10 (1.08-1.12) | <0.001 |  |
|  | 5–10 years | 1.12 (1.10-1.14) | <0.001 |  |
|  | >10 years | 1.16 (1.13-1.18) | <0.001 |  |
| **WHR, per SD** | Overall* | 1.15 (1.13-1.17) | <0.001 | <0.001 |
|  | 0–5 years | 1.12 (1.09-1.14) | <0.001 |  |
|  | 5–10 years | 1.15 (1.12-1.17) | <0.001 |  |
|  | >10 years | 1.21 (1.18-1.24) | <0.001 |  |
| **WHtR, per SD** | Overall* | 1.16 (1.15-1.17) | <0.001 | <0.001 |
|  | 0–5 years | 1.12 (1.09-1.14) | <0.001 |  |
|  | 5–10 years | 1.16 (1.14-1.18) | <0.001 |  |
|  | >10 years | 1.23 (1.20-1.26) | <0.001 |  |
| **ABSI, per SD** | Overall* | 1.06 (1.04-1.07) | <0.001 | <0.001 |
|  | 0–5 years | 1.01 (1.00-1.04) | 0.040 |  |
|  | 5–10 years | 1.06 (1.04-1.08) | <0.001 |  |
|  | >10 years | 1.13 (1.11-1.16) | <0.001 |  |

HRs are presented per 1-SD increment in each index. Model 1 was adjusted for age, sex, race, education, employment status, Townsend deprivation index, and assessment center. Model 2 was additionally adjusted for smoking status, drinking status, sleep duration, systolic blood pressure, depressive symptoms, coronary heart disease, stroke, diabetes, steroid use, and osteoarthritis.

**†**P values for temporal variation were obtained using likelihood ratio tests comparing the common-effect Cox model with the piecewise time-varying coefficient Cox model. *****Overall HRs were derived from conventional Cox proportional hazards models and represent average associations over the entire follow-up.

**Table S7.** Harrell’s C-index among six adiposity indices for incident back pain.

| **Obesity index** | **C-index (95% CI)** | **P-value vs. BMI** |
| --- | --- | --- |
| **BMI** | 0.6204 (0.6172, 0.6236) | -- |
| **Waist circumference** | 0.6203 (0.6171, 0.6235) | 0.9637 |
| **Hip circumference** | 0.6184 (0.6152, 0.6216) | 0.3940 |
| **WHR** | 0.6174 (0.6141, 0.6206) | 0.1932 |
| **WHtR** | 0.6201 (0.6169, 0.6234) | 0.9140 |
| **ABSI** | 0.6143 (0.6111, 0.6176) | 0.0095 |
